# Prenatal Diagnosis and Intervention in a Fetal Enterogenous Cyst

**DOI:** 10.7759/cureus.48208

**Published:** 2023-11-03

**Authors:** Shreya S Kushwaha, Upma Saxena, Poornima Sharma

**Affiliations:** 1 Obstetrics and Gynaecology, Vardhaman Mahavir Medical College and Safdarjung Hospital, New Delhi, IND

**Keywords:** intra-abdominal cyst, abdominal cyst, prenatal intervention, enterogenous cyst, enteric cyst

## Abstract

The ultrasound diagnosis of a fetal intra-abdominal cyst is typically established during the second or third trimester in the majority of cases. They primarily arise from the gastrointestinal or genitourinary system during the development of intra-abdominal structure and if isolated may resolve spontaneously. Enteric or enterogenous or enteric duplication cysts, which are congenital developments from the intestine, are most common. This case of an enterogenous cyst is presented because of its extreme rarity, its large size, and the need for prenatal intervention. Early identification and definitive treatment are necessary for proper management of this condition.

## Introduction

Despite being rare, the second and third-trimester ultrasound identification of a fetal abdominal cyst is a well-documented observation with potential implications for a diverse array of clinical and surgical issues. Within this context, the principal conditions to consider in the differential diagnosis encompass cystic formations that originate from the genitourinary tract, such as renal, ovarian, urachal, and adrenal cysts, as well as those originating from the gastrointestinal tract, which may include intestinal duplication cysts, mesenteric or omental cysts, hepatic or choledochal cysts, and dilated bowel loops due to atresia or obstruction [1,2]. Mesenteric cysts, simple hepatic cysts, ovarian cysts, choledochal cysts, intestinal duplication cysts, and meconium pseudocysts are more common conditions [3]. Isolated cysts observed during earlier stages of gestation are typically associated with favourable outcomes and often undergo spontaneous resolution [3,4]. The level of correlation between prenatal and postnatal diagnoses varies, ranging from 72.3% to 94.8% for fetuses presenting with abdominal cystic lesions [3,5].

Enteric duplication cysts can manifest at any location along the gastrointestinal tract, spanning from the oral cavity to the anus, and are frequently situated on the mesenteric side of the lumen [3,6]. Predominantly, these cysts tend to occur in the jejunum and ileum (53%), followed by colon (18%), duodenum (6%) or stomach (4%) [3,6]. Although the prevalence of duplications in general is not well understood, the overall incidence across various locations and types is likely around 1 in 5,000, even though any specific location or duplication type is uncommon [6]. These duplications can manifest in either cystic or tubular forms and have an outer layer of smooth muscle and a gastrointestinal epithelium lining on the inside and mucinous material within [6].

The majority of these abnormalities tend to remain asymptomatic during the prenatal phase, with symptoms typically manifesting at a later stage in postnatal life, often characterized by pain, bleeding, or intussusceptions [7]. Prenatal ultrasonography can be used to identify intestinal duplication cysts, allowing for early postnatal diagnosis [6]. Complementary to this, CT and MRI may be employed to precisely determine the location and size of the [6].

When a fetus is diagnosed with a cystic abdominal lesion, it is imperative to conduct a comprehensive ultrasound examination [3]. This not only aids in the precise characterization of the cyst but also helps to screen for possible associated anomalies [3]. Sometimes genitourinary system abnormalities, spinal cord duplications, hemivertebrae, and anterior myelomeningocele can coexist with enteric duplication [8,9]. They do not typically need fetal intervention and it is exceptionally required for very large cysts. The standard course of treatment generally entails surgical excision postnatally [6]. However, the specific location and extent of involvement may necessitate an individualized approach [6,7].

## Case presentation

**Figure 1:**
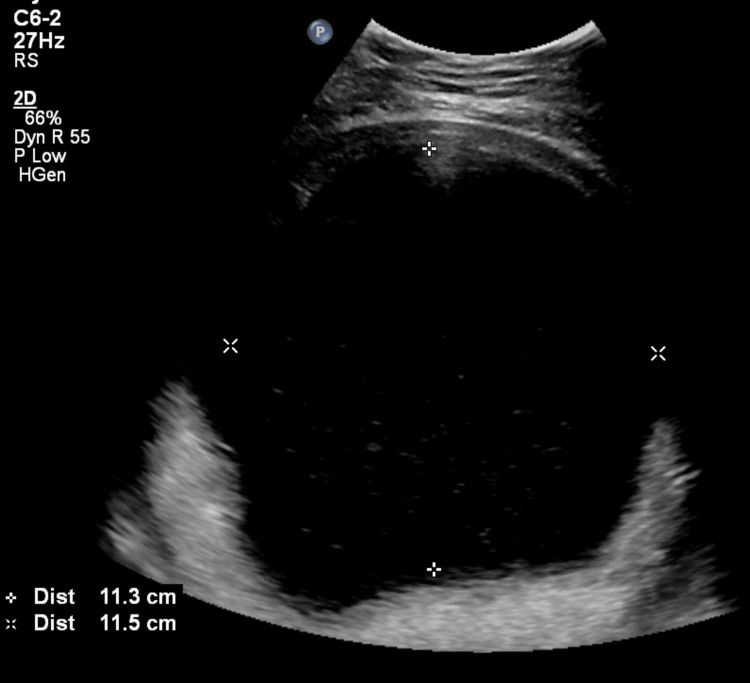
Grossly distended fetal abdomen

**Figure 2:**
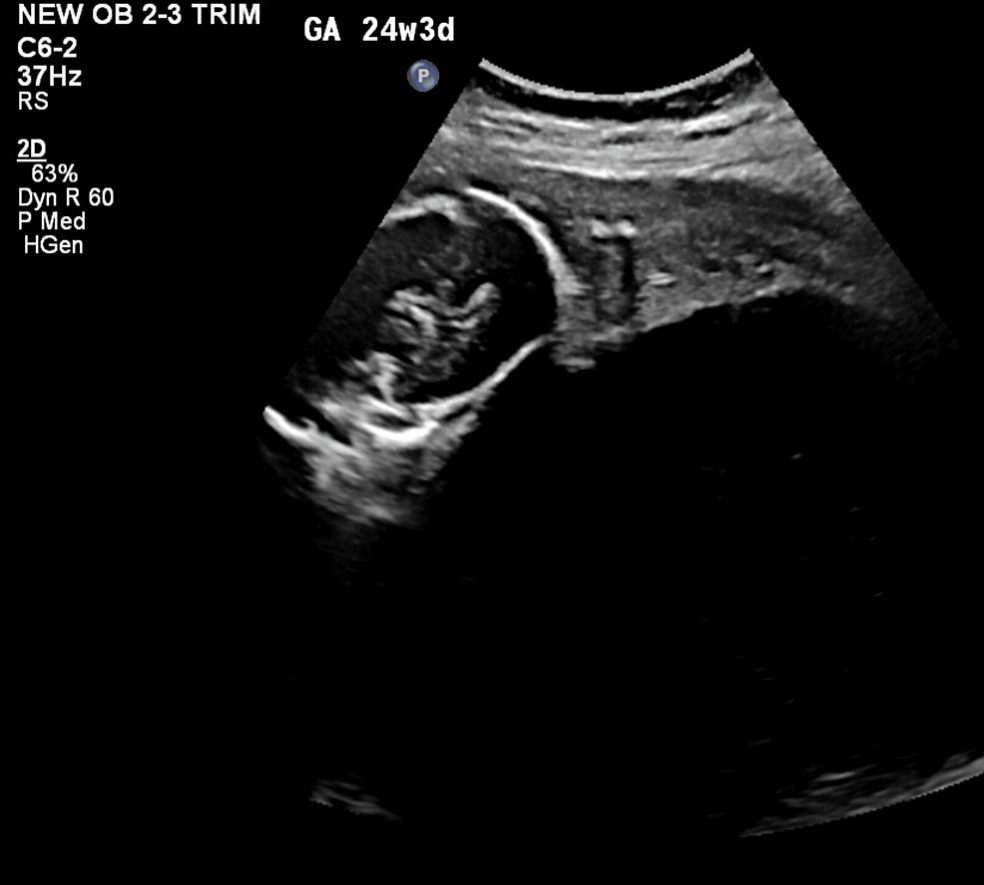
Fetus stuck to uterus

A 27-year-old woman, primigravida in a non-consanguineous marriage for three years, presented to fetal medicine OPD at 24+3 weeks with a fetal abdominal cyst. Her past and family history were insignificant. Her anomaly scans at 22+3 weeks, showed a single live intra-uterine fetus with a thin-walled cystic space occupying lesion (SOL) measuring 220 x 189 x 174 mm, arising from the fetal abdomen. The cyst was causing severe compression of the fetus; hence, fetal limbs, lower dorsal and lumbosacral spine and umbilical cord were not well visualized and the placenta was fundoposterior. A review scan at our centre showed the presence of a single intra-uterine fetus with cardiac activity, the fetus was stuck to the uterine wall with a grossly distended fetal abdomen and grossly distorted fetal anatomy (Figure 1 and Figure 2).

**Figure 3:**
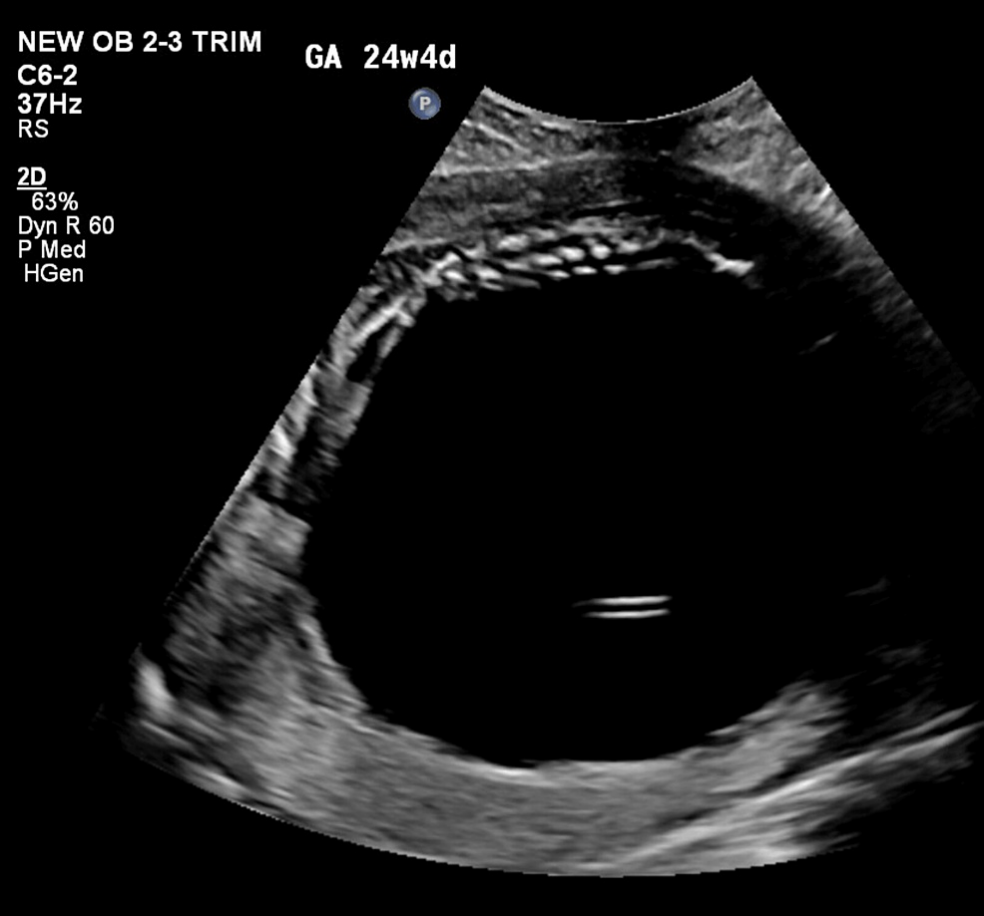
Aspiration being done

**Figure 4:**
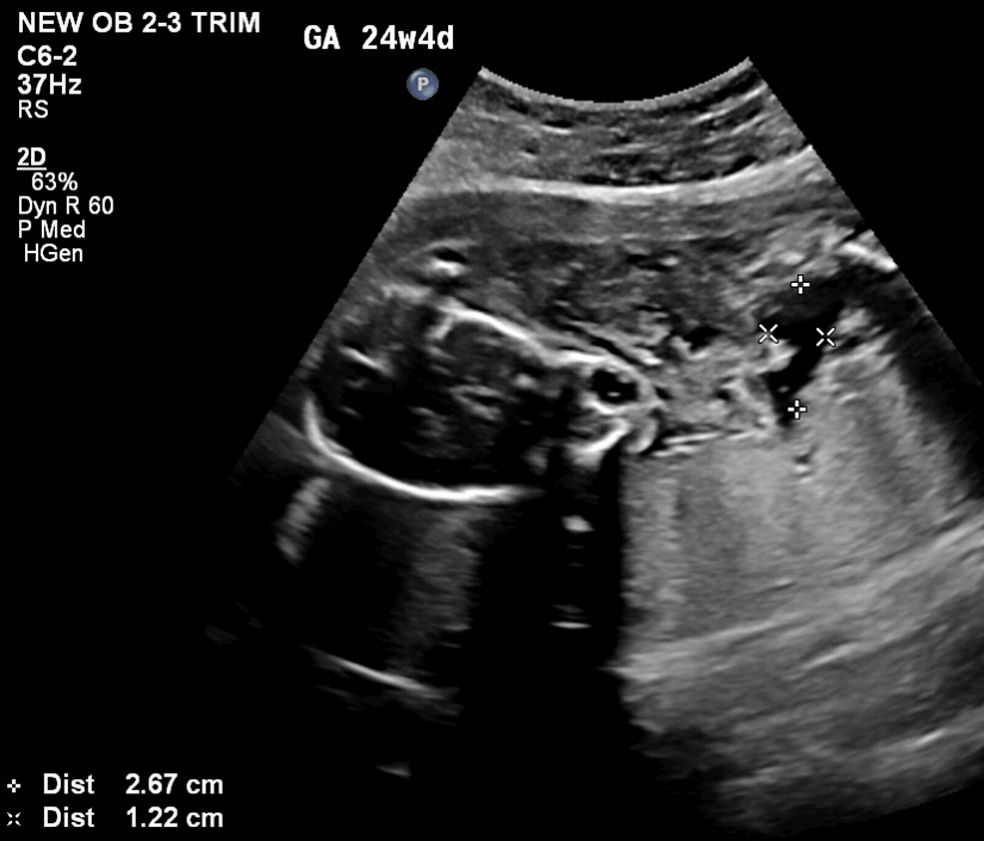
Post-aspiration USG

**Figure 5:**
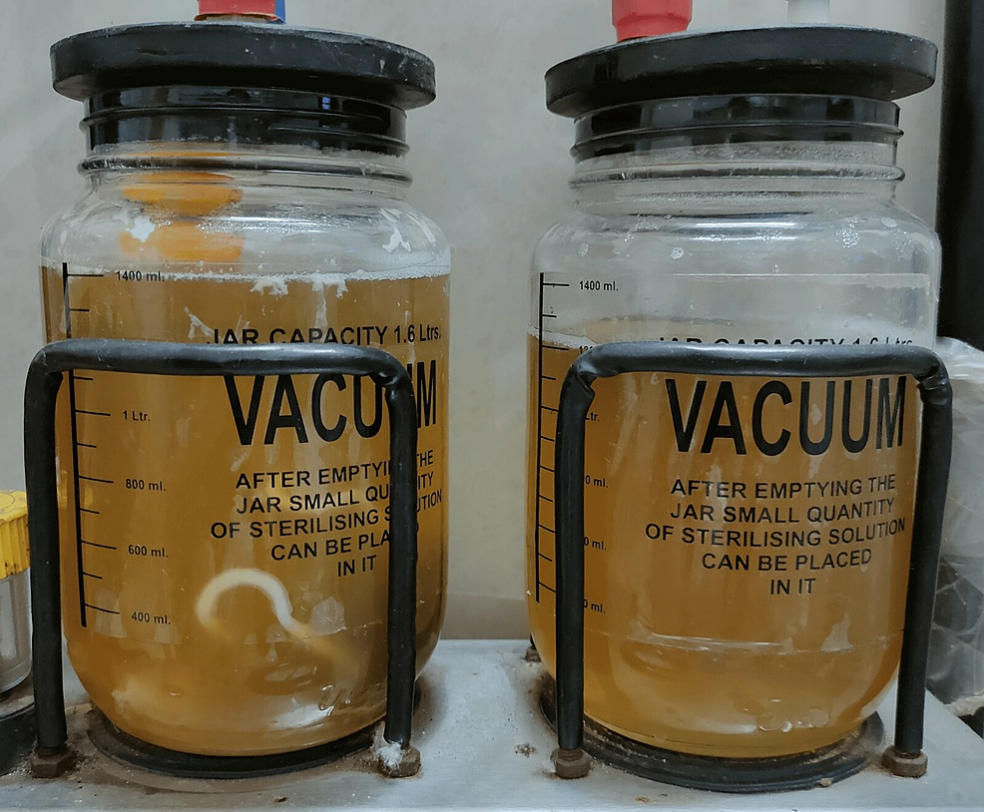
Aspirated fluid

She was counselled regarding the intra-abdominal cyst and the possibility of other anomalies as a detailed evaluation could not be done. She opted to discuss the issue with her family to decide on a further course of action. She returned the next day with a complaint of pain in her abdomen. On abdominal examination, the uterus corresponded to 28 weeks, and on per vaginal examination, the cervix was one finger tight and early effaced. A review scan was done that showed absent fetal cardiac activity. She was then planned for USG-guided abdominal decompression of the cyst (Figure 3 and Figure 4), which drained three litres of straw-coloured fluid (Figure 5).

**Figure 6:**
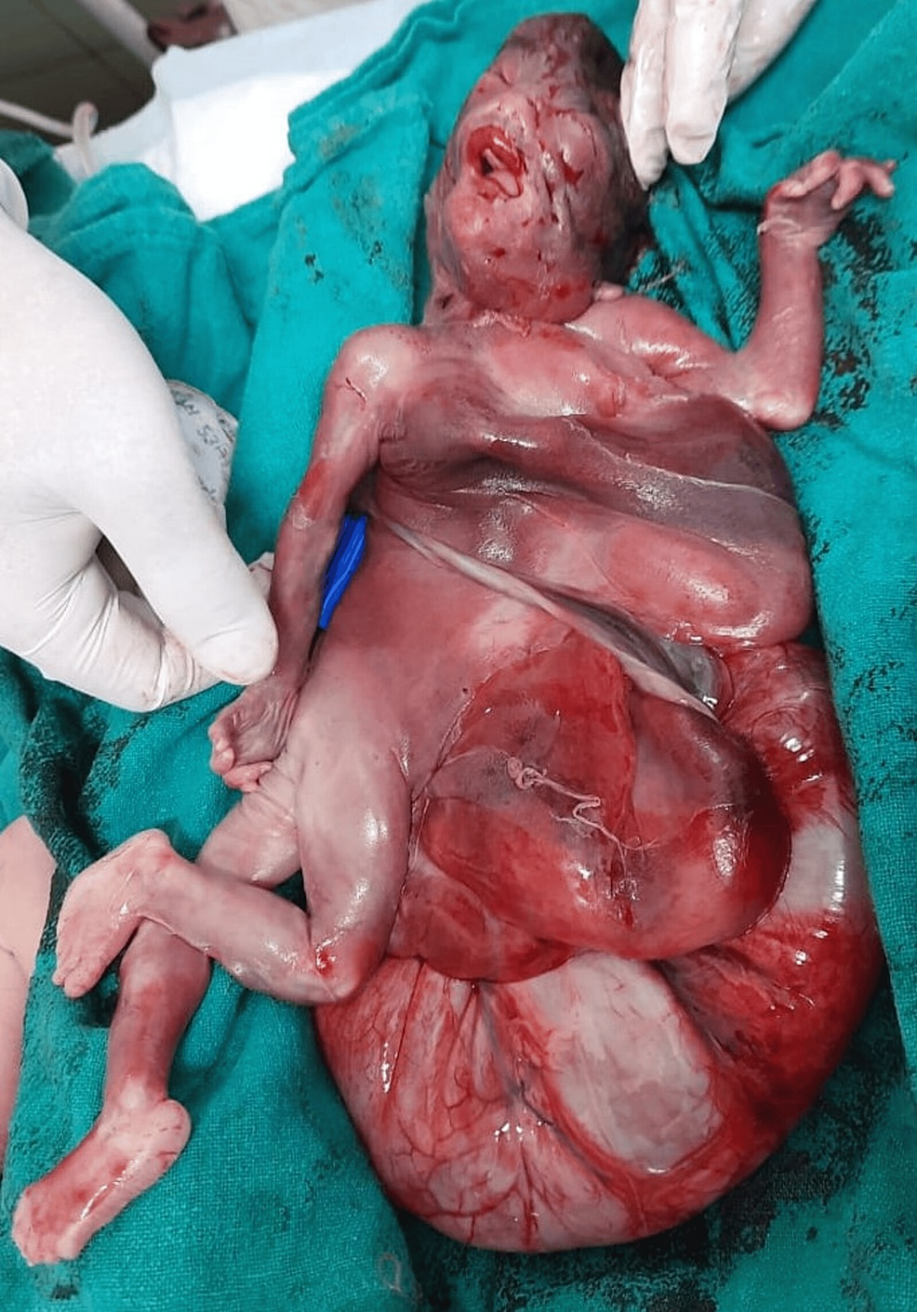
Expelled fetus (front)

**Figure 7:**
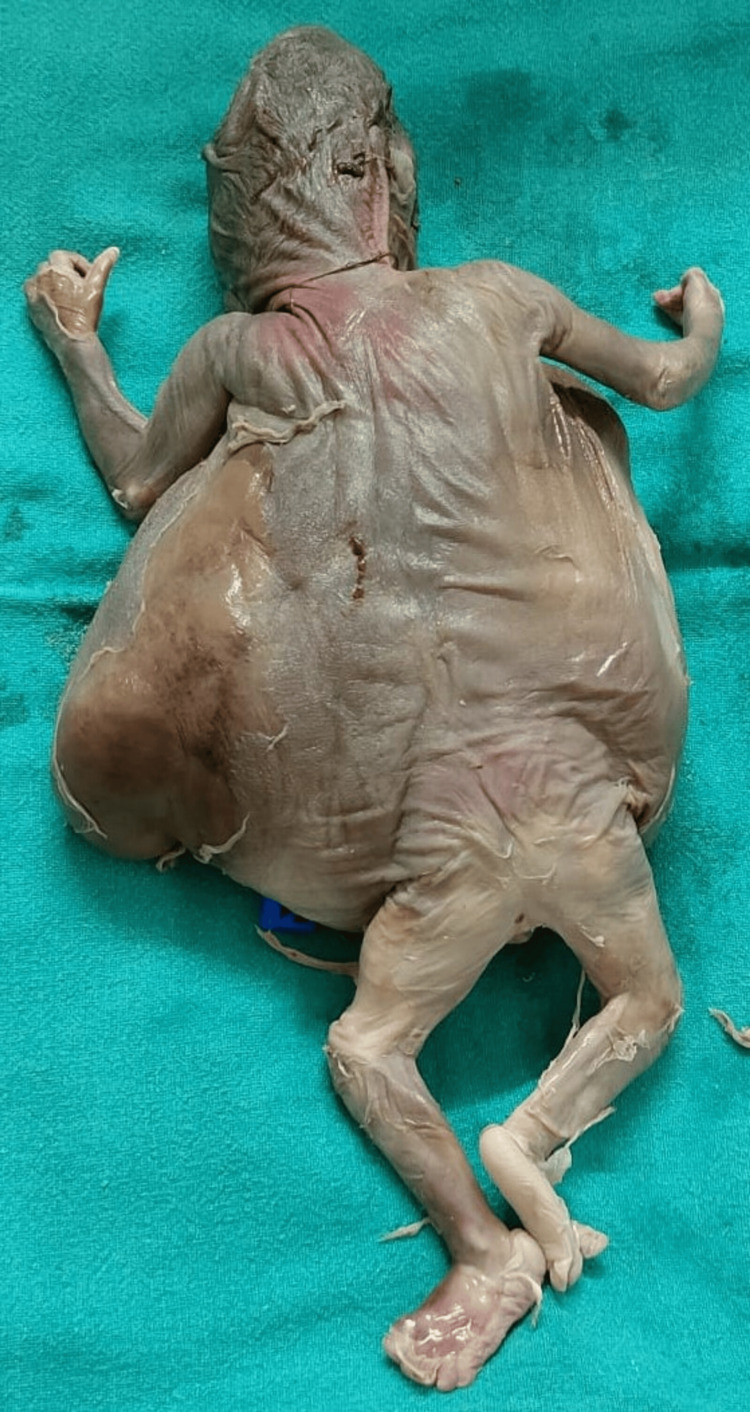
Expelled fetus (back)

Following this she went into spontaneous labour and delivered a macerated male baby of 1.2 kg. On gross examination, the fetal abdomen was massively distended (nearly 20x20 cm) but was not taut, as the fluid had already been drained (Figure 6 and Figure 7).

**Figure 8:**
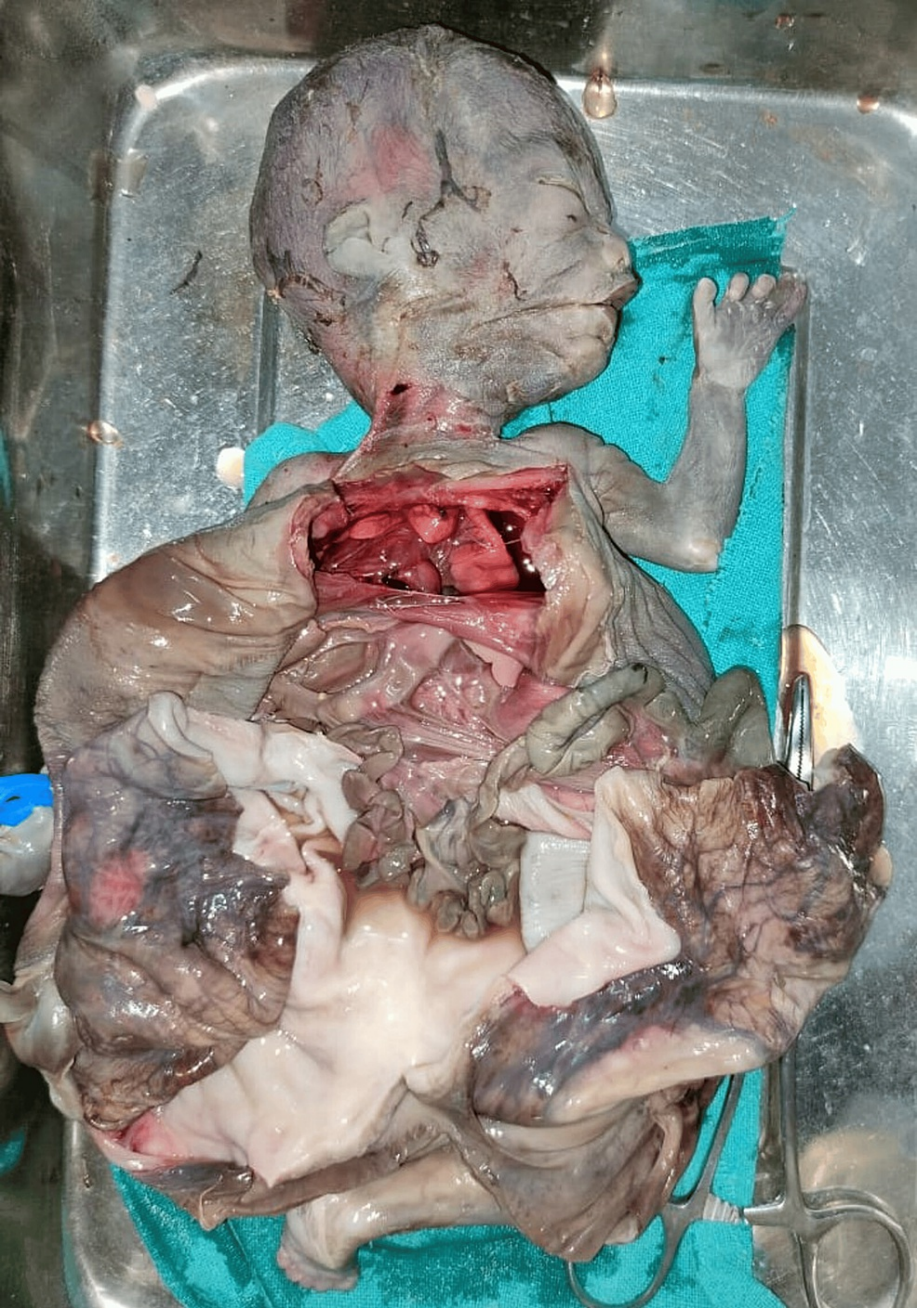
Autopsy pictures (inner surface of cyst)

**Figure 9:**
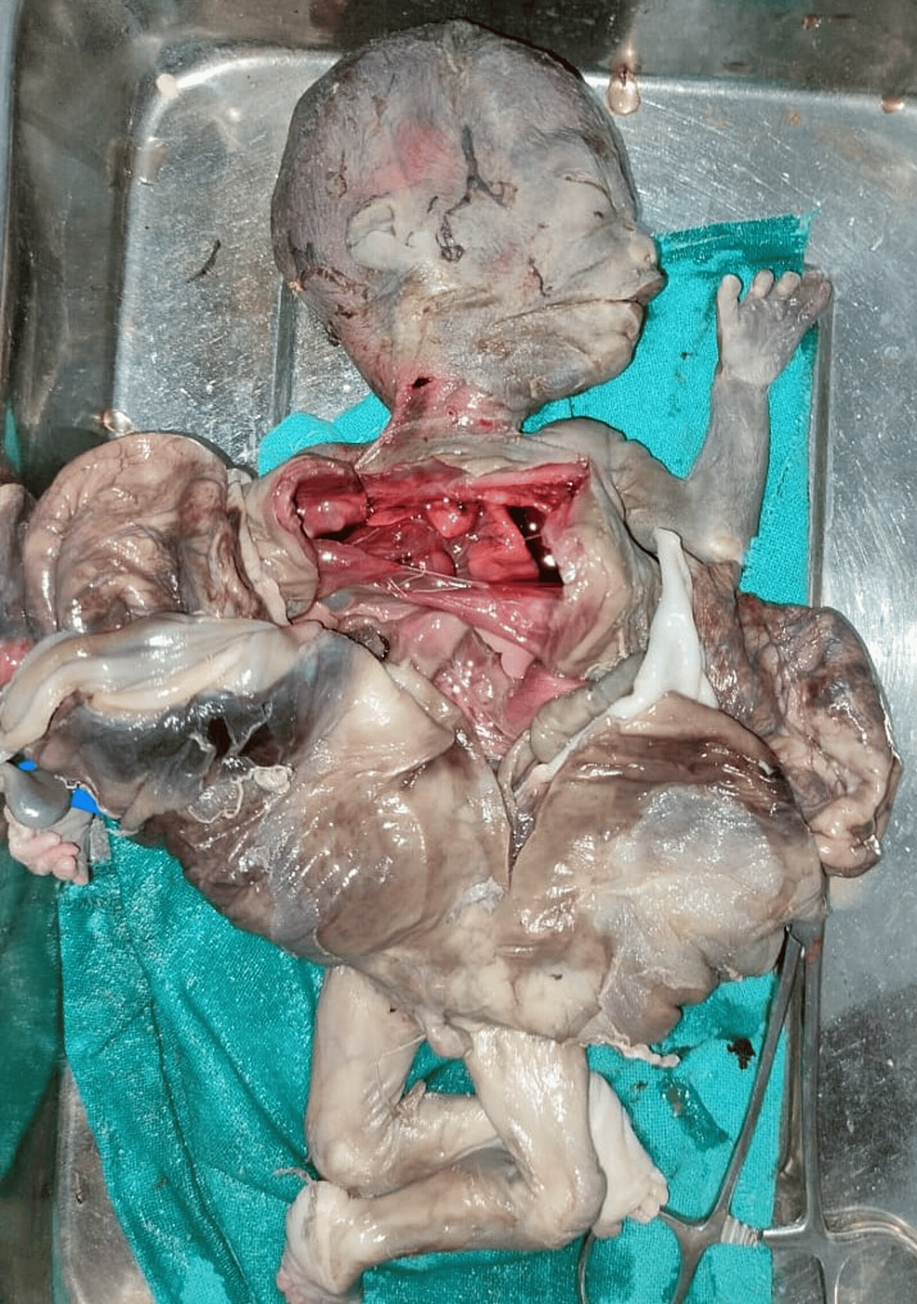
Autopsy pictures (outer surface of cyst)

On autopsy when the overlying covering was dissected a large cyst wall-like structure was seen 15x20 cm, arising from the mesentery of the intestine (Figure 8 and Figure 9).

Two reniform structures were identified near the retroperitoneum and other abdominal structures identified were grossly normal. The cyst wall and kidney-like structure were sent for histopathological examination. The cyst wall was reported as showing features of an enterogenous cyst and the specimen sent as a fetal kidney showed the presence of nephrogenic rests.

## Discussion

To the best of our knowledge, no previous case with such an enormous size has been described in the antenatal period. Also, we could not find such a case needing prenatal intervention for management. However, we could find important literature on the topic that we have discussed further. We hope that this adds to the information currently available.

Prenatal ultrasound screening stands as a valuable tool for evaluating intra-abdominal cystic lesions, yet it may not always provide an exact definitive diagnosis [10]. While the majority of cystic lesions can be treated conservatively and have good perinatal outcomes, certain cysts need to be surgically removed. Previous studies have indicated that an early gestational age at diagnosis and a smaller initial diameter are indicative of a likelihood of prenatal spontaneous regression [10]. Conversely, a larger cyst diameter detected before delivery predicts the necessity for postnatal surgical intervention [10]. Furthermore, a systematic review has corroborated that fetal abdominal cysts identified between 11 and 14 weeks of gestation are generally associated with positive perinatal outcomes and a tendency for spontaneous regression [4]. Lewis et al. retrospectively carried observational analysis of 38 fetal intra-abdominal cysts detected antenatally and reported that the maximal prenatal cyst diameter was helpful for the prediction of postnatal persistence of the cyst [8].

Hugele et al. reported 51% diagnostic accuracy of prenatal ultrasound for fetal intraabdominal cysts [11]. A recent study reported that MRI serves as a valuable supplementary tool to prenatal ultrasound, enhancing diagnostic accuracy with a reported rate of 73.4% [11]. However, studies have shown that cystic masses of the gastrointestinal tract can be accurately diagnosed in 91.1% of cases, using prenatal ultrasound [12].

According to a study by Sanna and coworkers on 80 fetal abdominal cysts, the majority were characterized as isolated (87.5%), located in the pelvic region (52%), classified as simple cysts (87.5%), and demonstrated avascularity (100%). During the antenatal period, 29% of these cysts resolved spontaneously, 29% reduced in size, 9% remained stable, and 33% increased in size. Among these cysts, 56% were of ovarian origin, followed by intestinal cysts. It was observed that 75% of cysts persisting postnatally necessitated surgical intervention. Notably, cysts originating from the intestine were often more challenging to diagnose antenatally and frequently required surgical management [13]. Another study by Ozyuncu et al. showed that out of 52 cases of abdominal cysts persisting postnatally, out of a total of 71 cases, nearly half of the cases required surgical correction [14].

An analysis of 68 publications, out of 1590 screened articles by Fahy et al. evaluated intestinal duplication cysts in 86 patients in which antenatal diagnoses were histologically confirmed postnatally. Out of these, 41% were resected within two days of age, and half of the infants diagnosed prenatally became symptomatic needing early resection. They observed that jejunal, proximal ileal, and colonic duplications were more frequently symptomatic [9].

## Conclusions

Abdominal cysts in early pregnancy tend to exhibit a propensity for spontaneous resolution or remaining at small sizes, typically resulting in favourable outcomes. However, serial ultrasound is advisable for close monitoring during the perinatal period. This is essential as abdominal cysts can occasionally be linked to more severe underlying gastrointestinal pathological conditions. Preterm labour and polyhydramnios are other risks. A multidisciplinary approach involving neonatologists and pediatric surgery will help to define the best place for delivery providing immediate access to neonatal surgical care. Need for prenatal intervention is required exceptionally if it is large, like the one in our instance.
